# A cluster-based approach for integrating clinical management of Medicare beneficiaries with multiple chronic conditions

**DOI:** 10.1371/journal.pone.0217696

**Published:** 2019-06-19

**Authors:** Brent M. Egan, Susan E. Sutherland, Peter L. Tilkemeier, Robert A. Davis, Valinda Rutledge, Angelo Sinopoli

**Affiliations:** 1 Care Coordination Institute, Prisma Health, Greenville, South Carolina, United States of America; 2 School of Medicine-Greenville, University of South Carolina, Greenville, South Carolina, United States of America; 3 Department of Medicine, Prisma Health Upstate, Greenville, South Carolina, United States of America; University of Groningen, NETHERLANDS

## Abstract

**Background:**

Approximately 28% of adults have ≥3 chronic conditions (CCs), accounting for two-thirds of U.S. healthcare costs, and often having suboptimal outcomes. Despite Institute of Medicine recommendations in 2001 to integrate guidelines for multiple CCs, progress is minimal. The vast number of unique combinations of CCs may limit progress.

**Methods and findings:**

To determine whether major CCs segregate differentially in limited groups, electronic health record and Medicare paid claims data were examined in one accountable care organization with 44,645 Medicare beneficiaries continuously enrolled throughout 2015. CCs predicting clinical outcomes were obtained from diagnostic codes. Agglomerative hierarchical clustering defined 13 groups having similar within group patterns of CCs and named for the most common CC. Two groups, congestive heart failure (CHF) and kidney disease (CKD), included 23% of beneficiaries with a very high CC burden (10.5 and 8.1 CCs/beneficiary, respectively). Five groups with 54% of beneficiaries had a high CC burden ranging from 7.1 to 5.9 (descending order: neurological, diabetes, cancer, cardiovascular, chronic pulmonary). Six groups with 23% of beneficiaries had an intermediate-low CC burden ranging from 4.7 to 0.4 (behavioral health, obesity, osteoarthritis, hypertension, hyperlipidemia, ‘other’). Hypertension and hyperlipidemia were common across groups, whereas 80% of CHF segregated to the CHF group, 85% of CKD to CKD and CHF groups, 82% of cancer to Cancer, CHF, and CKD groups, and 85% of neurological disorders to Neuro, CHF, and CKD groups. Behavioral health diagnoses were common only in groups with a high CC burden. The number of CCs/beneficiary explained 36% of the variance (R^2^ = 0.36) in claims paid/beneficiary.

**Conclusions:**

Identifying a limited number of groups with high burdens of CCs that disproportionately drive costs may help inform a practical number of integrated guidelines and resources required for comprehensive management. Cluster informed guideline integration may improve care quality and outcomes, while reducing costs.

## Introduction

In 2014, 42% of adults in the U.S. had multiple chronic conditions (CCs), defined as two or more comorbidities lasting at least a year, requiring recurrent medical attention and care, or limiting activities of daily living [1,2]. The prevalence of multiple CCs increases sharply with age, rising from 18% at ages 18–44 years to 81% in those 65 years and older [1]. Healthcare utilization and costs also rise rapidly as a function of the number of CCs. In 2014, hospital admissions increased from 6% of those with 1 or 2 CCs to 24% in those with five or more CCs, the number of prescriptions filled from 9 to 51 and the number of medical outpatient visits from 6 to 20. Twenty-eight percent of U.S. adults with ≥3 CCs accounted for two-thirds of healthcare costs, whereas the 72% with 0–2 CCs accounted for one-third [1].

In 2001, the Institute of Medicine noted that patients with multiple CCs often failed to receive care for one or more CCs leading to suboptimal outcomes [3]. The 2001 Report recommended integrated clinical guidelines. Yet, guidelines remain focused largely on single CCs, despite repeated recognition of their limitations and the importance of integration [4–8].

Primary care clinicians are burdened with implementing individual disease management guidelines. Estimates indicated that a typical fulltime primary care clinician would work 21.7 hours daily to implement all recommended care and 10 hours for the 10 most common CCs [9, 10]. A systematic analysis of 20 evidence-based guidelines for four CCs found that attention to comorbid conditions was limited, the quality of the evidence often moderate to low, and that specific treatment recommendations for comorbidities were often lacking [6]. Patients with multiple CCs reported receiving conflicting instructions and suboptimal care coordination, and the authors recommended that future guidelines address these limitations.

One of many challenges in developing integrated clinical guidelines for multiple CCs is the number of different combinations. To risk adjust payments, the Centers for Medicare and Medicaid Services (CMS) uses hierarchical condition categories (HCCs), which are mainly CCs. In 2008, 32% of Medicare beneficiaries had two or more HCCs, including >2,000,000 unique combinations, and accounted for 79% of costs [11]. Developing integrated guidelines for even a small percentage of >2,000,000 unique HCC groups is impractical.

Using an exploratory multi-dimensional clustering analysis [12], we sought to determine if a limited number of groups, based on similar within group patterns of CCs, could be identified in a relatively large group of Medicare beneficiaries. This unsupervised mode of learning was chosen rather than a modeling-based approach, since we were interested in discerning if patterns in the data could be detected given the absence of a priori knowledge of patient subgroups. If homogeneous subgroups of patients with chronic conditions were found, these findings could serve as a starting point for developing integrated clinical guidelines for groups of patients with similar patterns of CCs. Such an approach could promote more efficient and comprehensive management, while favorably impacting the patient experience, care outcomes and costs.

## Methods

### Regulatory considerations

Data from the Electronic Health Records Systems (EHRS) were obtained from patient encounters in our Accountable Care Organizations (ACO) and linked with the Medicare Shared Savings Program (MSSP) assignment and the limited claim and claim line feed (CCLF) data for 2015. All personal identifiers were removed prior to analysis. This study of secondary data analysis was reviewed and approved with waiver of consent in accordance with 45 CFR 46.116 and use of protected health information under 45 CFR 164.512 by the Greenville Hospital System Institutional Review Board Committee A, IRB# 00000805, operating under Federal Wide Assurance #00001380 for the Protection of Human Subjects. Initial approval of the study was issued on August 29, 2017, and renewed on an annual basis thereafter.

### Patient inclusion criteria and data

The analysis file included all MSSP beneficiaries continuously assigned to the ACO in 2015 who were 27 years or older with EHRS data and who had one or more diagnoses from ICD-9-CM or ICD-10-CM codes in 2015 or in any prior year. Patients < 65 years old were included since individuals in this age group who receive Social Security Disability Insurance benefits qualify for Medicare benefits [13]. EHRS data abstracted included age, sex, race, body mass index, smoking status, number of outpatient visits, and diagnoses.

Thirty-seven distinct categories of CCs were constructed for each patient from diagnostic codes in the EHRS and the primary diagnosis from inpatient hospitalizations in 2015. Inclusion of any of the 37 distinct CCs in a discrete or structured diagnostic field of the EHRS or on the CCLF for inpatient claim type was accepted as evidence for presence of the CC [14]. More specifically, recent evidence indicates that capturing diagnoses in EHRS over several years is more likely to approximate expected rates of prevalence for CCs than a one-year period [14].

Thirty-one diagnoses were first categorized according to the enhanced Elixhauser coding algorithm [15]. Patients with a body mass index (BMI) ≥30 kg/m^2^ from the EHRS were included in the obese category. Three selected additional CCs defined by Charlson comorbidities, myocardial infarction, cerebrovascular disease and dementia, were included [15]. The remaining three conditions, coronary atherosclerosis and heart disease, lipid metabolism disorders, and osteoarthritis were added using diagnoses codes defined in the Clinical Classifications Software (CCS) of the Healthcare Cost and Utilization Project (HCUP) [16]. The average number of outpatient visits during three years (2013–2015) was used to quantify visits for each patient on an annual basis rather than using one year in order to provide a stable estimate of the number of usual visits.

### Analysis

An agglomerative hierarchical clustering approach was chosen because it does not require the number of clusters to be pre-specified and it results in each patient being assigned to one and only one group. The process starts with all patients in individual clusters and progressively joins patients who are most similar at each step until there is only one cluster at the conclusion. Nesting of clusters allows the researcher to choose the number of clusters based on statistical criteria and preferences at any step in the process [17]. The overall arching framework for this analysis was to determine if a manageable number of meaningful clusters could be identified for subsequent development of clinical guidelines.

Each chronic condition was coded as absent or present, i.e., 0 or 1, for each patient. Selected CCs were combined, reducing the number of categories from 37 to 25, which was done primarily to facilitate the objective of identifying a limited set of clinical important and distinct clusters. For example, complicated and uncomplicated hypertension were combined into a single category of hypertension (HTN), and similarly complicated and uncomplicated diabetes (DM) were combined into one category since the designation ‘complicated’ is based on co-morbid conditions, attributable to organ systems captured in the data. Lymphoma, metastatic cancer and solid tumors without metastasis were combined into a single category for cancer (CA) as these conditions signify a requirement for an oncologist; coronary atherosclerosis, myocardial infarction, cerebrovascular disease and peripheral vascular disease were combined into a cardio-vascular group (CVD). These cardiovascular diagnoses identify a group at very high risk for additional vascular events, which benefits from comprehensive risk factor management [18]. Dementia, paralysis and other neurological disorders identify CCs within the neurology domain (Neuro), and the behavioral health (BH) group was comprised of depression, psychoses, drug abuse and alcohol abuse to identify a group for whom co-management with a specialist in behavioral health/ psychiatry is appropriate [19]. Finally, 12 chronic disease groups representing 24 diagnostic categories were chosen for clustering, primarily based on a prevalence ≥15%, association with clinical management by different medical specialists or subspecialists, and their relevance for this project. Some CCs with a prevalence >15% were not included in the cluster analysis given their close relationship to conditions often co-managed either by primary care or the same subspecialty clinicians as those in the cluster analysis (e.g., arrhythmias with congestive heart failure [CHF] or coronary heart disease; fluid and electrolyte disorders with CHF or chronic kidney disease [CKD]). All 12 chronic conditions were considered to be of equal importance and the correlation coefficients between all pairs examined to avoid over-representation of any one condition [20].

The analysis file was randomly divided into three unique patient subgroups (S1 and S2 Tables) and sorted by number of CCs in descending order. Clustering methods were employed for each subgroup in an independent manner, using SAS Enterprise Guide software, version 7.1 [21]. The only variables included in the clustering model were the binomial indicators for the 12 selected CCs. Cluster groups were developed in several steps. First, a distance matrix was computed between all patient pairs, using Jaccard’s distance measure with the asymmetric option that considers the presence of CCs more important than their absence [21, 22]. The resulting dissimilarity matrix was used as the input into agglomerative hierarchical clustering models using Ward’s minimum variance method [22, 23]. Inspection of local maximum values of the pseudo F and local minimum values from pseudo t-squared statistics suggested several clustering solutions [24–26]. A cluster size of 13 was chosen, based upon consistency across subgroups (S1 and S2 Figs), the prevalence distribution of CCs within each cluster (S3–S5 Tables), and the goal of identifying a feasible number of clusters for informing integrated clinical guidelines [27, 28]. Similar cluster profiles from each of the three subgroups were used to map patients to a common cluster assignment (S6 Table). Cluster assignments were then merged with clinical and claims data for each patient. The coefficient of determination, R^2^, was determined from Pearson’s correlation coefficient of the relationship between number of CCs and total claims paid per beneficiary.

### Data reporting

Each of the 13 clusters was named for the most prevalent CC within that cluster. Demographic and clinical data from the EHRS were summarized for patients in each cluster. The average number of out-patient department (OPD) visits in the previous three years, 2013–2015, was determined.

Paid claims, the amount paid by Medicare (Medicare “costs”) were summed for each patient with a claim starting in 2015, from Parts A and B, and included Durable Medical Equipment (DME) services. Average total costs from all encounters, including acute and chronic conditions, per patients in each cluster were calculated. Claims with a type indicating an inpatient claim in Part A were used to identify hospitalization events. Claims data were available for >99% of beneficiaries since claims were unavailable for those who declined to share their data or for claims related to medications or drug and alcohol treatment [29].

## Results

In this Medicare beneficiary cohort, 85% were ≥65 years, 88% White and 58% female with an average of 6.3 CCs and 4 healthcare visits annually (Table 1). Beneficiaries who were < 65 years of age were more likely to be Black and Male, had higher BMI values, and were much more likely to be current smokers. Both age groups had the same number of CCs. Blacks were younger than Whites, and they had a higher BMI and a slightly greater number of CCs. Women were approximately two years older than men, and they were less likely to report smoking.

**Table 1:** Descriptive characteristics of Medicare beneficiaries with age, race, sex subgroups.

		Age		Race		Sex	
Variable	All	<65	≥65	Black	White	Men	Women
**N (%)**	44,645 (100)	6861(15.4)	37,784 (84.6)	4181 (9.8)	37,385 (87.5)	18,374 (42.3)	25,034 (57.7)
**Age, years**	71.7 ± 0.05	53.3 ± 0.11	75.0 ± 0.04	67.0 ± 0.20	72.3 ± 0.05	70.8 ± 0.08	72.3 ± 0.07
**Race: White, %**	87.5	73.9	89.9			88.2	86.9
**Race: Black, %**	9.8	22.3	7.6			9.1	10.3
**Race: Other, %**	2.8	3.8	2.6			2.7	2.8
**Male: Female, %**	42.3 : 57.7	46.2 : 53.8	41.6 : 58.4	39.1 : 60.9	42.6 : 57.4		
**OPD Visits/year**	4.0 ± 0.02	4.6 ± 0.05	3.9 ± 0.02	4.0 ± 0.06	4.0 ± 0.02	3.9 ± 0.03	4.0 ± 0.03
**BMI, kg/m^2^**	28.8 ± 0.03	31.2 ± 0.09	28.3 ± 0.03	30.7 ± 0.11	28.6 ± 0.03	29.1 ± 0.04	28.5 ± 0.04
**Current Smoker, %**	22.3	43.9	18.3	27.2	21.7	25.2	20.1
**CCs**^a^	6.3 ± 0.02	6.3 ± 0.04	6.3 ± 0.02	6.7 ± 0.05	6.4 ± 0.02	6.3 ± 0.03	6.5 ± 0.02

Data are presented as mean ± standard error of the mean or percent; Abbreviations: OPD, outpatient department; BMI, body mass index; CCs, chronic conditions

Missing Data: Race (1896), Sex (1237), BMI (2576), Smoking (8796);

^a^ Based on 25 categories of CCs

Table 2 lists the prevalence of 25 CCs among the Medicare beneficiaries with some CCs including multiple diagnoses. Patients with more than one diagnosis in a single category were counted only once. Hypertension and lipid disorders were the most prevalent conditions, affecting 8 of 10 persons, followed by cardiovascular disease (CVD), osteoarthritis, and obesity. Behavioral health diagnoses, chronic pulmonary disease (including asthma) (CPD), diabetes, and cardiac arrhythmias each affected about one-third of beneficiaries. Hypothyroidism and fluid and electrolyte disorders affected roughly one-fourth of patients. Various cancers, valvular (heart) disease, neurological conditions, chronic kidney disease (renal failure) and congestive heart failure were prevalent in more than 15% of beneficiaries. Deficiency anemias, weight loss, rheumatoid arthritis/collagen vascular and liver diseases affected one of seven to ten patients. The remaining CCs affected <10% of patients.

**Table 2:** Prevalence of chronic conditions in 44,645 Medicare beneficiaries.

Chronic Condition	N	Percent
Hypertension*	36,260	81.2
Lipid Metabolism Disorders*	35,297	79.1
Cardiovascular Disease*	20,757	46.5
Osteoarthritis*	20,483	45.9
Obesity*	17,971	40.3
**Behavioral Health** *	15,716	35.2
Chronic pulmonary disease*	15,068	33.8
Diabetes*	14,910	33.4
**Cardiac Arrhythmias**	13,930	31.2
**Hypothyroidism**	12,407	27.8
**Fluid and Electrolyte Disorders**	11,755	26.3
Cancer*	8,758	19.6
**Valvular Disease**	7,917	17.7
Neurological Conditions*	7,501	16.8
Chronic Kidney Disease*	7,378	16.5
Congestive Heart Failure*	7,200	16.1
**Deficiency Anemia**	5,985	13.4
**Weight Loss**	4,940	11.1
**Rheumatoid Arthritis/collagen**	4,641	10.4
**Liver Disease**	4,459	10.0
**Pulmonary Circulation Disorders**	3,261	7.3
**Coagulopathy**	2,476	5.6
**Peptic Ulcer Disease excluding bleeding**	1,804	4.0
**Blood Loss Anemia**	1,425	3.2
**AIDS/HIV**	308	0.7

*Chronic Condition included in cluster analysis

Twelve CCs reflecting 24 diagnostic categories were selected to develop clusters. Thirteen patient clusters were identified with each group labeled according to its most prevalent CC (Table 3). Roughly 2% of beneficiaries had no recorded diagnosis for the 12 CCs selected for clustering. The mean number of CCs was 6.3 of 25 categories (Table 2) with a range of 0 to 24.

**Table 3:** Prevalence (%) of chronic conditions in 13 clusters subdivided by burden of CCs.

CC Burden	Very High		High					Low to Intermediate					
	CHF	CKD	Neuro	CA	CVD	CPD	DM	BH	Obes	OA	HTN	HLP	Other
**N**	6044	4053	4176	5099	5730	4125	5093	3024	1879	1873	1736	794	1019
**Pts, %**	13.5	9.1	9.4	11.4	12.8	9.2	11.4	6.8	4.2	4.2	3.9	1.8	2.3
**CCs**^a^**, n**	10.5	8.1	7.4	6.5	6.1	5.9	5.9	4.7	3.6	3.4	2.5	1.6	0.4
**HLP**	89.7	87.3	73.8	79.5	88.7	75.8	84.6	68.2	69.5	69.5	70.0	**100.0**	0.0
**HTN**	96.3	95.6	80.9	80.0	87.6	78.3	87.6	68.6	72.8	65.2	**100.0**	0.0	0.0
**OA**	56.3	50.7	42.6	44.3	45.8	51.9	48.7	39.3	36.2	**100.0**	0.0	0.0	0.0
**Obes**	55.6	39.8	30.4	32.3	44.1	41.1	49.3	39.4	**100.0**	15.1	0.0	0.0	0.0
**BH**	50.2	33.3	50.7	30.1	30.9	26.3	35.5	**99.8**	0.0	0.0	0.0	0.0	0.0
**DM**	54.7	44.7	29.8	20.1	28.9	17.9	**99.2**	2.6	0.0	0.0	0.0	0.0	0.0
**CPD**	59.9	33.2	34.5	28.9	22.4	**99.4**	22.3	22.2	0.0	0.0	0.0	0.0	0.0
**CVD**	80.7	59.1	58.7	42.7	**99.8**	38.6	23.0	8.4	6.4	0.0	0.0	0.0	0.0
**CA**	23.2	17.8	10.9	**100.0**	2.3	12.6	8.1	0.5	0.0	0.0	0.0	0.0	0.0
**Neuro**	26.9	15.8	**97.5**	10.9	4.0	0.9	6.2	0.9	0.0	0.0	0.0	0.0	0.0
**CKD**	36.5	**99.9**	12.6	7.0	0.5	2.4	1.6	1.0	0.0	0.0	0.0	0.0	0.0
**CHF**	**94.8**	13.2	10.9	6.1	0.9	1.1	0.8	1.0	0.0	0.0	0.0	0.0	0.0

^a^ Based on the 25 conditions shown in Table 2

Highest prevalence of the CC in each cluster is shown in bold

Abbreviations: CC(s) chronic condition(s); N, number; HLP, lipid metabolism disorders; HTN, hypertension; OA, osteoarthritis; Obes, obesity; BH; CVD, cardiovascular disease; CPD, chronic pulmonary disease; CA, cancer; DM, diabetes; Neuro, neurological conditions); CKD, chronic kidney disease; CHF, congestive heart failure

The very high, high and intermediate to low-burden groupings were based on the percentage of patient with six or more CCs. In the very high burden group, >80% of patients had six or more CCs, in the high burden group >50 to < 75% of patients had six or more CCs and in the intermediate to low-burden groups, fewer than one-third of patients had six or more CCs (Tables 3 and 4). Two cluster groups, CHF and CKD, comprised ~23% of beneficiaries and had the highest burden of CCs. Five clusters with 54% of beneficiaries had a high burden of CCs: neurological conditions (Neuro), diabetes (DM), cancers (CA), chronic pulmonary disease (CPD) and cardiovascular disease (CVD). The remaining 23% of beneficiaries had an intermediate to low burden of CCs: behavioral health (BH), obesity (Obes), osteoarthritis (OA), hypertension (HTN), lipid metabolism disorders (hyperlipidemia, HLP), and others.

**Table 4:** Clinical characteristics of the 13 clusters subdivided by burden of chronic conditions.

CC Burden	Very High		High					Low to Intermediate					
	CHF	CKD	Neuro	CA	CVD	CPD	DM	BH	Obes	OA	HTN	HLP	Others
**Age, years**	74.0±.1	74.6±.2	71.4±.2	73.6±.1	72.6±.1	70.7±.2	69.6±.2	65.6±.2	68.2±.2	73.0±.2	72.7±.2	70.0±.3	68.8±.4
** <65 years, %**	15	12	24	8	11	16	20	32	16	7	6	7	18
** ≥65 years, %**	85	88	76	92	89	85	80	68	84	94	94	93	82
**White:Black,%**	84 : 12	79 : 15	83 : 10	80 : 7	88 : 7	87 : 7	78 : 14	84 : 8	82 : 11	88 : 6	83 : 6	90 : 2	71 : 6
**Other:Unk, %**	2 : 3	2 : 4	2 : 5	2 : 2	2 : 3	3 : 3	4 : 5	3 : 5	4 : 4	3 : 4	4 : 8	3 : 4	3 : 19
**Female,%**	53	51	60	53	51	63	58	72	58	70	63	64	59
**OPD Visits/yr**	5.9±.1	4.9±.1	4.3±.1	4.0±.1	3.8±.1	3.9±.1	3.6±.1	3.8±.1	2.4±.1	2.7±.1	1.9±.1	1.6±.1	1.0±.1
** 0–1, %**	22	24	29	28	30	33	34	29	44	40	51	58	78
** ≥4, %**	60	56	48	46	44	43	43	45	26	32	17	12	6
**CCs**^a^ **≤ 2, %**	0.3	1	5	5	3	7	4	11	22	26	57	87	98
**CCs**^a^ **≥ 6, %**	94	82	71	60	56	52	52	31	11	8	1	0.1	0.2
**BMI, kg/m^2^**	30.1±.1	28.8±.1	27.3±.1	27.8±.1	29.1±.1	28.7±.1	30.5±.1	28.6±.1	33.5±.1	26.5±.1	25.3±.1	24.8±.1	24.4±.1
**≥ 1 Hosp, %**	35	24	27	18	13	12	12	9	4	9	3	1	3

Data are presented as either percent or mean ± standard error

Abbreviations: OPD, number of outpatient visits; CC, Chronic Conditions; BMI, body mass index; Hosp, hospital inpatient admission; NA = not applicable or none available; Number Missing Data: Race (1896); Sex (1237), OPD visits (1068), BMI (1738)

^a^ Based on the 25 conditions shown in Table 2

Demographic, clinical and hospitalization data are summarized for each cluster as shown in Table 4. The youngest clusters were BH, Obes, and others, whereas the CKD and CHF clusters had the highest ages. While the overall ratio of men to women was about 2:3, the sex differential was greatest in the BH and OA clusters and least in the CKD and CVD clusters. The number of outpatient visits was greatest in the two highest burden clusters (CHF, CKD), and least in five intermediate to low burden clusters (Obes, OA, HTN, HLP, others). More patients were hospitalized in the CHF (35%), Neuro (27%) and CKD (24%) as compared to the other clusters. Less than 10% of patients in the Low to Intermediate Burden groups had a hospitalization.

**Fig 1:**
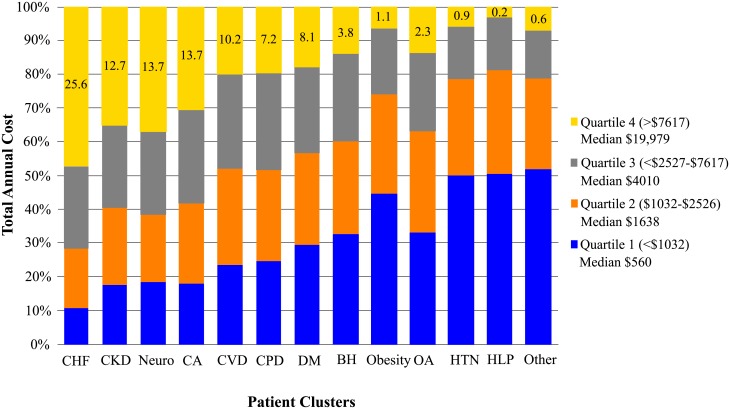
Quartiles of Medicare paid claims for each cluster. The distribution of the total claims paid (cost) is determined by quartile. The percentage of patients represented in the top quartile of cost generally align with the number of CCs per cluster, which are greatest for clusters on the left and decrease progressively in moving from left to right. Numbers within the yellow bars reflect percentages of all patients in the upper quartile of cost for each cluster group.

**Fig 2:**
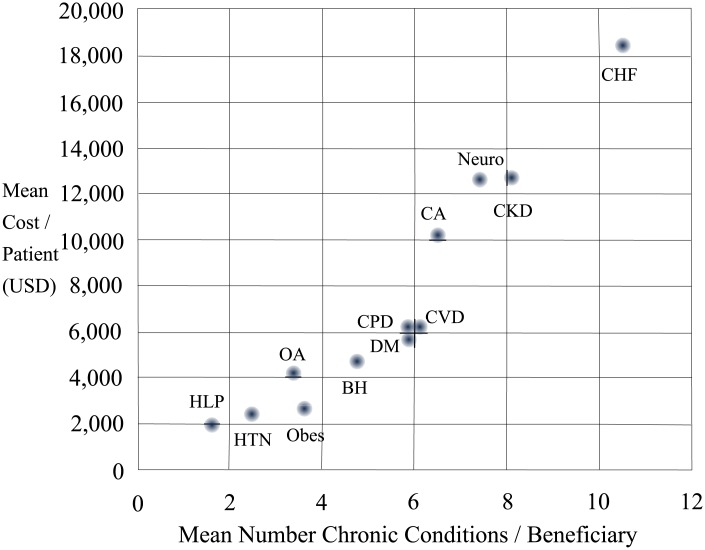
Relationship between mean number of CCs and mean paid claims across 12 groups of Medicare beneficiaries. The mean number of CCs per group is shown on the horizontal axis and the mean total of Medicare claims paid per group is depicted on the y-axis. Abbreviations for each group are the same as in Table 3.

The cost of Medicare claims paid in 2015 for this cohort was $394,855,871, which excluded claims for medications and diagnoses such as alcohol and drug use disorders. The mean of CMS paid claims per beneficiary was $8,844. Quartiles of US dollar values of claims paid were determined for all beneficiaries, and the percentage of patients within each quartile are shown by cluster (Fig 1). Patients in the CHF and CKD clusters combined accounted for more than 38% of top quartile cost, while patients in the five high burden clusters combined for another 53% of top costs. Conversely, the six low to intermediate clusters accounted for 38% of beneficiaries in the lowest quartile of cost. Mean CMS paid claims per beneficiary across clusters aligned closely with mean numbers of CCs per cluster (Fig 2).

## Discussion

Multiple CCs are common in the U.S. population, especially in older groups including Medicare beneficiaries [1,2]. Among 44,645 Medicare beneficiaries who were continuously enrolled in one accountable care organization during 2015, 95% had two or more CCs (Table 2). A prior report indicated that 68% of Medicare beneficiaries had two or more CCs from diagnoses on paid claims [30]. The prevalence of CCs in this group of Medicaid beneficiaries based on EHRS information is greater than in a prior report, which used CMS paid data. Our finding is consistent with studies that documented a higher prevalence of chronic conditions from EHRS than claims data [31]. The difference may also reflect the greater burden of CCs in the Southeast than other U.S. regions [32]. The prevalence of chronic conditions reflected diagnoses contained in the EHRS, since reliable data were available and evidence indicates that estimates of chronic condition prevalence are more accurate when several years of data are used rather than a single year [14].

Considerable overlap in CCs was seen between cluster groups (Table 3). Hypertension and disorders of cholesterol metabolism were the second and third most prevalent conditions in nine clusters with the largest burden of CCs, which serve to highlight limitations of condition specific guidelines. Yet, several important CCs segregate disproportionately to a limited number of clusters, which has important implications for developing integrated clinical guidelines. For example, 80% of beneficiaries with a CHF diagnosis segregated to the CHF cluster, 85% with CKD segregated to the CKD and CHF clusters, 85% with neurological disorders (Neuro, CHF, and CKD) and 82% with cancer (Cancer, CHF, CKD) segregated to three clusters. Moreover, 88% of beneficiaries with diabetes segregated to four (DM, CHF, CKD, CVD) and 88% with chronic pulmonary disease to five (CPD, CHF, Cancer, Neuro, CKD) clusters.

In the CHF and CKD cluster groups, >80% of patients had six or more chronic conditions, which was defined as a very high burden of chronic disease. In fact, all 12 chronic conditions representing 24 diagnoses included in the cluster analysis, occurred with a frequency of >15% in these two groups. From a practical perspective, it may be prudent and efficient to have a subspecialist readily available at the point of care when the prevalence of chronic conditions within their expertise is present in 15% or more of patients with multiple CCs.

In the Neurological (Neuro), Cancer (CA), Cardiovascular (CVD), Chronic Pulmonary Disease (CPD), and Diabetes (DM) clusters, >50% but <75% had six or more chronic conditions. Moreover, <15% of patients in the Neuro group had CHF or CKD, indicating that referral to these specialists could be considered rather than having their expertise available at the point of care. Yet, inclusion of Cardiology and Nephrology in developing an integrated guideline for patients within the high burden of disease clusters could lead to better management of these conditions within primary care and fewer but more appropriate referrals. In the Cancer group, <15% of patients had Neurological, CKD or CHF diagnoses, whereas in the CVD, CPD, and DM groups, <15% of patients had diagnoses indicating cancer, neurological disorders, CKD, and CHF. As noted above, inclusion of these specialties, despite a prevalence of <15% for each of their discipline-specific diagnoses could be beneficial.

In the six clusters with an intermediate-low burden of CCs, fewer than one-third of patients had six or more chronic conditions. In fact, when excluding the behavioral health cluster, the other five clusters had fewer than 15% of individuals with six or more chronic conditions. Of note, except for the behavioral health cluster, these five low-burden clusters had no diagnoses of behavioral health conditions in their EHRS data. Moreover, in these groups, the prevalent conditions are appropriately managed within primary care with little, if any, requirement for co-management by subspecialists.

In developing an integrated guideline, it would be logical to include the most common CC and other commonly occurring CCs in each group. The integrated guideline could (i) identify high-level, evidence-based recommendations for the most common CC with notation of recommendations beneficial for comorbid CCs, (ii) inform the subspecialists and other medical personnel required to co-manage each cluster group together with primary care, (iii) highlight evidence-based recommendations for one or more CC that are contra-indicated for concomitant CCs. This item is especially important as the EHRS may fail to alert users of drug-disease interactions or generate excessive low-value alerts leading to user fatigue and oversight of important drug-disease interactions [33–34], (iv) provide guidance on what levels of disease severity are generally appropriate for primary care clinicians to manage and specific referral criteria, (v) inform more complete and congruent patient education, and (v) streamline care management and transition plans.

Behavioral health diagnoses were common in patients with a high burden of CCs but essentially absent in those with a low burden. A similar finding was reported from a cluster analysis of patients with two or more CCs in the Kaiser Permanente Colorado health maintenance organization [35]. The presence of behavioral diagnoses in patients with multiple CCs identifies a subset more likely to require emergency department and inpatient services, which amplify costs [36]. The strong linkage of behavioral health diagnoses with multiple CCs in Medicare beneficiaries highlights a largely unmet need to effectively integrate behavioral health and medical care. Of note, women constitute an overwhelming majority of patients in the behavioral health cluster, where a focus on women’s health appears appropriate.

Most patients in the seven complex clusters had four or more outpatient visits yearly, which provide opportunities to improve care quality and outcomes. Unfortunately, healthcare delivery and outcomes are suboptimal for patients with multiple CCs. The Institute of Medicine’s 2001 Report noted that important evidence-based healthcare services may not be provided, while care that is provided often includes non-essential or low-value services [3, 37]. Subsequent reports reiterated and expanded upon the call for integrated guidelines for patients with multiple CCs in the IOM 2001 Report [4–6]. Yet, progress in publishing integrated clinical guidelines for patients with multiple CCs is limited [4–8]. The principal focus has remained on disease management programs for specific CCs [38]. Moreover, recommendations for one CC can adversely affect other comorbid conditions. For example, medications including citalopram for depression, which prolong the QT intervals increase risk for sudden death, especially in at-risk patients including those with heart failure and long QT syndrome [33, 39].

In this patient cohort, the number of CCs accounted for 36% of the variance (R^2^ = 0.36) in Medicare paid claims, which is consistent with previous reports [1, 2, 11, 30]. Moreover, the linear relationship between the mean number of CCs and mean costs per beneficiary in each cluster is evident (Fig 2). Disease-specific management programs have improved targeted health outcomes and can reduce costs [40]. However, there is substantial heterogeneity and some comprehensive disease-management programs have worsened outcomes [41–43]. Disease-specific management for military veterans with severe CPD reduced hospital and emergency department admissions for CPD and cardiac causes [44]. However, hospital and emergency department admissions for other diagnoses were not reduced and comprised a significant proportion of all admissions. This report is concordant with another analysis, which found that annual healthcare costs were $4,040 higher for patients with than without CPD [45]. After adjustment for demographic variables and concomitant diseases, the annual excess costs for CPD declined to $520. These data support the notion that effective management of comorbid conditions, which could be facilitated by integrated guidelines, is important.

Study limitations include reliance on diagnostic codes in EHRS and paid claims, limited information on condition severity, and incomplete Medicare paid claims data. Diagnoses may be incomplete and, in some cases, erroneous. Our analysis did not include disease severity or social determinants, which are important modifiers of outcomes and costs. Our data are, nonetheless, consistent with reports that healthcare costs rise in tandem with the number of CCs in Medicare beneficiaries [1, 30].

CHF and CKD, which were associated with the most comorbid CCs in our analysis, are also linked with very high annual healthcare costs [46]. Another limitation is that various clustering algorithms can produce varying results. The occurrence of ties in computing similarity measures presents an additional challenge in cluster analysis (S7 Table), particularly when a relatively small number of dichotomous variables are used to form homogenous groups in a large cohort. While our initial clustering analysis in Medicare beneficiaries in one ACO generated consistent cluster patterns in three randomly allocated groups, additional work is required to determine similarities and differences with alternative clustering approaches applied to various elderly clinical populations.

Our principal finding is that seven clusters of patients representing approximately three-fourths of Medicare beneficiaries had a very high or high burden of CCs, including a substantial minority with behavioral health diagnoses. Our findings suggest that the cluster groups may serve to inform a limited number of integrated clinical guidelines for the principal and common comorbid diagnoses. The integrated guideline could also include guidance on the resources and expertise as well as the patient education and support resources required for integrated, comprehensive management of each cluster. Our analysis addresses one of several barriers to integrated guidelines, i.e., the number of unique combinations of CCs [11]. Despite limitations, application of the conceptual approach outlined in this report could potentially provide an important starting point for a more integrated approach to improving care quality and outcomes at lower cost.

## Supporting information

S1 Table Number and prevalence of chronic conditions for randomly assigned patient groups. ^a^p-value from Chi-Square statistic; no adjustment for multiple comparisons. Abbreviations: N, number; HTN, hypertension (complicated and/or uncomplicated); DM, diabetes mellitus (complicated and/or uncomplicated); Obesity (diagnosis and/or BMI ≥30 kg/m2); Cancer (solid tumor without metastasis, metastatic cancer, lymphoma); CVD, cardiovascular diseases (coronary disease, peripheral vascular disorders, cerebrovascular disease, myocardial infarction); Behavioral, behavioral health (depression, alcohol abuse, drug abuse, psychoses); Lipid, lipid metabolism disorders; OA, osteoarthritis; CPD, chronic pulmonary disease; CKD, chronic kidney disease including renal failure; CHF, congestive heart failure; Neuro, neurological conditions (dementia, paralysis, other neurological disorders).
(DOCX)

S2 Table Relative frequency of number of chronic conditions by randomly assigned patient group. ^a^ Chronic Conditions defined by diagnostic codes for 12 disease categories used to develop patient clusters; no statistically significant difference in the distribution across 3 groups (χ^2^, p = 0.4571). ^b^ All patients randomly assigned to one of 3 patient groups (A,B or C).
(DOCX)

S3 Table Number and prevalence of chronic conditions in 13 clusters for patients randomly assigned to group A. Abbreviations: HTN, hypertension; OA, osteoarthritis; CVD, cardiovascular disease; CPD, chronic pulmonary disease; CKD, chronic kidney disease; CHF, congestive heart failure.
(DOCX)

S4 Table Number and prevalence of chronic conditions in 13 clusters for patients randomly assigned to group B. Abbreviations: HTN, hypertension; OA, osteoarthritis; CVD, cardiovascular disease; CPD, chronic pulmonary disease; CKD, chronic kidney disease; CHF, congestive heart failure.
(DOCX)

S5 Table Number and prevalence of chronic conditions in 13 clusters for patients randomly assigned to group C. Abbreviations: HTN, hypertension; OA, osteoarthritis; CVD, cardiovascular disease; CPD, chronic pulmonary disease; CKD, chronic kidney disease; CHF, congestive heart failure.
(DOCX)

S6 Table Number and prevalence of chronic conditions in 13 clusters for all 3 patient groups combined. Cluster assignment in each randomly assigned group is shown in top 3 rows with mapping to the aggregated cluster in row 4. Cluster names in row 4 are assigned based on the most prevalent chronic condition in each of the 12 aggregated clusters. Abbreviations: HTN, hypertension; OA, osteoarthritis; CVD, cardiovascular disease; CPD, chronic pulmonary disease; CKD, chronic kidney disease; CHF, congestive heart failure; DM, diabetes mellitus; CA, cancer; BH, behavioral health; Obes, obesity.
(DOCX)

S7 Table Prevalence of chronic conditions by cluster assignment for six permutations of order in patient subgroup A. To evaluate the effect of ties in the distance data, the clustering analysis is repeated for several random permutations of patient order. Permutations are created by random re-ordering of patients, using those in subgroup A. The prevalence of conditions among patients in each cluster are shown here for the repeated analysis. Permutation #1 contains the results of patients in the order previously described in the paper, and permutations # 2 through # 6 are shown for five additional random orders. The cluster numbers are arranged for convenience in comparing the results. There is complete agreement in 3 clusters for all permutations, shown in the 3 last columns and highlighted in blue. Two additional clusters show identical results from permutations 2, 3 and 6 which are highlighted in yellow. Other examples where the prevalence of a particular condition is identical or differs slightly (<3%) in each permutation is highlighted in grey. For other chronic conditions, there is often close agreement in 5 of the 6 permutations. Overall, there is general agreement in the clustering solutions, especially when viewed in terms of burden of disease. However, there are differences that remain. Further analysis with various clustering methods and/or with additional populations may provide insight and confirmation of these results. Abbreviations: HTN, hypertension; OA, osteoarthritis; CPD, chronic pulmonary disease; CVD, cardiovascular disease; CKD, chronic kidney disease; CHF, congestive heart failure.
(DOCX)

S1 Fig Pseudo t^2^ by number of clusters for 3 independent patient groups. Plot of the pseudo t^2^ statistic for cluster sizes 5 to 20 from independent agglomerative clustering of 3 partitions (A, B, C) of the patient cohort. The pseudo t^2^ statistic, a measure of the separation between the 2 most recently joined clusters, peaks at 12; suggesting a cluster size of 13 in all 3 groups.
(EPS)

S2 Fig Pseudo F statistic by number of clusters for 3 independent patient groups. Plot of pseudo F statistic for cluster sizes 5 to 20 from independent agglomerative clustering of 3 partitions (A,B, C) of the patient cohort. The pseudo F statistic is a measure of the separation among all clusters at the current level. The relatively large value of the statistic suggests a stopping point at 13 clusters in all 3 groups of patients.
(EPS)
