# Supplementary material for: A cluster-based approach for integrating clinical management of Medicare beneficiaries with multiple chronic conditions
Source: PLoS One. 2019 Jun 19;14(6):e0217696. doi: 10.1371/journal.pone.0217696 (PMC6584004; doi:10.1371/journal.pone.0217696)
Supplement: S1 Table — ap-value from Chi-Square statistic; no adjustment for multiple comparisons. Abbreviations: N, number; HTN, hypertension (complicated and/or uncomplicated); DM, diabetes mellitus (complicated and/or uncomplicated); Obesity (diagnosis and/or BMI ≥30 kg/m2); Cancer (solid tumor without metastasis, metastatic cancer, lymphoma); CVD, cardiovascular diseases (coronary disease, peripheral vascular disorders, cerebrovascular disease, myocardial infarction); Behavioral, behavioral health (depression, alcohol abuse, drug abuse, psychoses); Lipid, lipid metabolism disorders; OA, osteoarthritis; CPD, chronic pulmonary disease; CKD, chronic kidney disease including renal failure; CHF, congestive heart failure; Neuro, neurological conditions (dementia, paralysis, other neurological disorders). (DOCX) [file pone.0217696.s001.docx]

|  | **Patient Group** | | |  |
| --- | --- | --- | --- | --- |
|  | **A** | **B** | **C** | **p-value^a^** |
| N | 14,970 | 14,973 | 14,702 |  |
| HTN | 80.86 | 81.80 | 80.99 | 0.0788 |
| DM | 33.46 | 33.79 | 32.93 | 0.2911 |
| Obesity | 40.23 | 40.90 | 39.61 | 0.0777 |
| Cancer | 19.55 | 19.50 | 19.81 | 0.7748 |
| CVD | 46.38 | 46.17 | 46.94 | 0.3900 |
| Behavioral | 34.94 | 35.44 | 35.23 | 0.6537 |
| Lipid | 79.06 | 79.22 | 78.90 | 0.7929 |
| OA | 45.64 | 46.22 | 45.78 | 0.5793 |
| CPD | 34.21 | 33.39 | 33.66 | 0.3089 |
| CKD | 16.53 | 16.87 | 16.17 | 0.2722 |
| CHF | 15.97 | 16.19 | 16.22 | 0.8157 |
| Neuro | 17.09 | 16.72 | 16.58 | 0.4757 |
