# Supplementary material for: A cluster-based approach for integrating clinical management of Medicare beneficiaries with multiple chronic conditions
Source: PLoS One. 2019 Jun 19;14(6):e0217696. doi: 10.1371/journal.pone.0217696 (PMC6584004; doi:10.1371/journal.pone.0217696)
Supplement: S2 Table — a Chronic Conditions defined by diagnostic codes for 12 disease categories used to develop patient clusters; no statistically significant difference in the distribution across 3 groups (χ2, p = 0.4571). b All patients randomly assigned to one of 3 patient groups (A,B or C). (DOCX) [file pone.0217696.s002.docx]

|  | **Patient Group**^b^ | | |
| --- | --- | --- | --- |
| **Number Chronic Conditions**^a^ | **A** | **B** | **C** |
| 0 | 2.29 | 2.16 | 2.40 |
| 1 | 5.60 | 5.26 | 5.28 |
| 2 | 9.67 | 9.90 | 9.86 |
| 3 | 14.90 | 15.35 | 14.71 |
| 4 | 17.19 | 16.95 | 17.88 |
| 5 | 16.61 | 16.30 | 16.56 |
| 6 | 12.87 | 13.29 | 13.06 |
| 7 | 9.89 | 9.28 | 9.41 |
| 8 | 5.89 | 5.89 | 5.52 |
| 9 | 2.89 | 3.21 | 3.10 |
| 10 | 1.53 | 1.55 | 1.50 |
| 11 | 0.53 | 0.68 | 0.54 |
| 12 | 0.15 | 0.17 | 0.18 |
