# Supplementary material for: A cluster-based approach for integrating clinical management of Medicare beneficiaries with multiple chronic conditions
Source: PLoS One. 2019 Jun 19;14(6):e0217696. doi: 10.1371/journal.pone.0217696 (PMC6584004; doi:10.1371/journal.pone.0217696)
Supplement: S3 Table — Abbreviations: HTN, hypertension; OA, osteoarthritis; CVD, cardiovascular disease; CPD, chronic pulmonary disease; CKD, chronic kidney disease; CHF, congestive heart failure. (DOCX) [file pone.0217696.s003.docx]

| **Cluster Number** | **A1** | **A2** | **A4** | **A6** | **A3** | **A7** | **A5** | **A8** | **A9** | **A10** | **A11** | **A12** | **A13** |
| --- | --- | --- | --- | --- | --- | --- | --- | --- | --- | --- | --- | --- | --- |
| Patients, N | 2034 | 1667 | 1300 | 1674 | 1487 | 1072 | 2141 | 1067 | 805 | 508 | 579 | 293 | 343 |
| Patients, % | 13.6 | 11.1 | 8.7 | 11.2 | 9.9 | 7.2 | 14.3 | 7.1 | 5.4 | 3.4 | 3.9 | 2.0 | 2.3 |
| **Chronic Conditions, %** |  |  |  |  |  |  |  |  |  |  |  |  |  |
| Lipid Metabolism Disorders | 90.5 | 88.1 | 72.2 | 82.1 | 78.8 | 74.4 | 89.0 | 66.1 | 72.4 | 69.1 | 70.0 | 100.0 | 0.0 |
| HTN | 95.8 | 95.6 | 79.2 | 87.2 | 80.6 | 72.7 | 87.3 | 66.8 | 75.3 | 64.8 | 100.0 | 0.0 | 0.0 |
| OA | 57.8 | 48.4 | 47.8 | 41.7 | 45.9 | 46.2 | 44.3 | 45.7 | 50.8 | 100.0 | 0.0 | 0.0 | 0.0 |
| Obesity | 53.1 | 35.8 | 34.2 | 44.9 | 38.3 | 28.6 | 47.3 | 43.1 | 100.0 | 0.0 | 0.0 | 0.0 | 0.0 |
| Behavioral Health | 50.5 | 36.4 | 44.9 | 32.5 | 29.0 | 2.9 | 44.1 | 99.5 | 0.0 | 0.0 | 0.0 | 0.0 | 0.0 |
| CVD | 81.6 | 63.2 | 49.8 | 20.9 | 38.9 | 36.2 | 100.0 | 0.4 | 15.0 | 0.0 | 0.0 | 0.0 | 0.0 |
| CPD | 60.1 | 35.2 | 33.8 | 28.7 | 25.0 | 99.8 | 28.2 | 32.7 | 0.0 | 0.0 | 0.0 | 0.0 | 0.0 |
| Cancer | 27.2 | 23.7 | 16.3 | 3.0 | 100.0 | 15.6 | 2.2 | 1.3 | 0.0 | 0.0 | 0.0 | 0.0 | 0.0 |
| Diabetes | 55.5 | 43.9 | 29.7 | 100.0 | 22.9 | 0.5 | 34.5 | 0.6 | 0.0 | 0.0 | 0.0 | 0.0 | 0.0 |
| Neurological Conditions | 26.2 | 24.3 | 99.9 | 3.7 | 4.8 | 0.3 | 8.3 | 0.9 | 0.0 | 0.0 | 0.0 | 0.0 | 0.0 |
| CKD | 33.5 | 99.8 | 1.9 | 1.8 | 2.2 | 1.9 | 1.0 | 0.0 | 0.0 | 0.0 | 0.0 | 0.0 | 0.0 |
| CHF | 94.7 | 17.3 | 5.5 | 0.5 | 2.1 | 0.5 | 1.5 | 2.8 | 0.0 | 0.0 | 0.0 | 0.0 | 0.0 |
