# Supplementary material for: A cluster-based approach for integrating clinical management of Medicare beneficiaries with multiple chronic conditions
Source: PLoS One. 2019 Jun 19;14(6):e0217696. doi: 10.1371/journal.pone.0217696 (PMC6584004; doi:10.1371/journal.pone.0217696)
Supplement: S4 Table — Abbreviations: HTN, hypertension; OA, osteoarthritis; CVD, cardiovascular disease; CPD, chronic pulmonary disease; CKD, chronic kidney disease; CHF, congestive heart failure. (DOCX) [file pone.0217696.s004.docx]

| **Cluster Number** | **B1** | **B2** | **B6** | **B4** | **B3** | **B8** | **B5** | **B7** | **B10** | **B9** | **B11** | **B12** | **B13** |
| --- | --- | --- | --- | --- | --- | --- | --- | --- | --- | --- | --- | --- | --- |
| Patients, N | 2205 | 1290 | 1028 | 2210 | 2000 | 730 | 1882 | 1194 | 451 | 831 | 586 | 243 | 323 |
| Patients, % | 14.7 | 8.6 | 6.9 | 14.8 | 13.4 | 4.9 | 12.6 | 8.0 | 3.0 | 5.5 | 3.9 | 1.6 | 2.2 |
| **Chronic Conditions, %** |  |  |  |  |  |  |  |  |  |  |  |  |  |
| Lipid Metabolism Disorders | 90.9 | 85.8 | 67.8 | 85.9 | 82.0 | 66.9 | 87.5 | 71.4 | 66.1 | 70.5 | 68.8 | 100.0 | 0.0 |
| HTN | 97.1 | 95.8 | 76.9 | 89.3 | 80.9 | 71.9 | 88.0 | 70.8 | 69.6 | 67.9 | 100.0 | 0.0 | 0.0 |
| OA | 55.3 | 54.4 | 34.1 | 55.5 | 46.7 | 57.0 | 47.9 | 28.5 | 0.0 | 100.0 | 0.0 | 0.0 | 0.0 |
| Obesity | 55.8 | 42.8 | 27.8 | 51.4 | 30.9 | 37.3 | 46.0 | 36.3 | 100.0 | 33.9 | 0.0 | 0.0 | 0.0 |
| Behavioral Health | 57.0 | 31.2 | 48.3 | 37.1 | 29.7 | 25.8 | 19.0 | 100.0 | 0.0 | 0.0 | 0.0 | 0.0 | 0.0 |
| CVD | 82.3 | 55.0 | 52.3 | 35.4 | 46.4 | 3.0 | 99.3 | 20.9 | 0.0 | 0.0 | 0.0 | 0.0 | 0.0 |
| CPD | 63.3 | 29.7 | 28.7 | 21.9 | 35.7 | 100.0 | 36.0 | 26.9 | 0.0 | 0.0 | 0.0 | 0.0 | 0.0 |
| Cancer | 20.5 | 9.4 | 2.7 | 10.9 | 100.0 | 0.3 | 4.0 | 0.0 | 0.0 | 0.0 | 0.0 | 0.0 | 0.0 |
| Diabetes | 54.2 | 47.8 | 19.0 | 98.3 | 20.9 | 0.1 | 20.9 | 5.5 | 0.0 | 0.0 | 0.0 | 0.0 | 0.0 |
| Neurological Conditions | 34.2 | 16.7 | 100.0 | 10.2 | 11.0 | 0.4 | 2.7 | 0.5 | 0.0 | 0.0 | 0.0 | 0.0 | 0.0 |
| CKD | 39.3 | 100.0 | 4.0 | 1.9 | 14.2 | 0.0 | 0.2 | 0.0 | 0.0 | 0.0 | 0.0 | 0.0 | 0.0 |
| CHF | 91.8 | 11.1 | 2.2 | 1.3 | 9.3 | 0.0 | 1.0 | 0.0 | 0.0 | 0.0 | 0.0 | 0.0 | 0.0 |
|  |  |  |  |  |  |  |  |  |  |  |  |  |  |
