# Supplementary material for: A cluster-based approach for integrating clinical management of Medicare beneficiaries with multiple chronic conditions
Source: PLoS One. 2019 Jun 19;14(6):e0217696. doi: 10.1371/journal.pone.0217696 (PMC6584004; doi:10.1371/journal.pone.0217696)
Supplement: S5 Table — Abbreviations: HTN, hypertension; OA, osteoarthritis; CVD, cardiovascular disease; CPD, chronic pulmonary disease; CKD, chronic kidney disease; CHF, congestive heart failure. (DOCX) [file pone.0217696.s005.docx]

| **Cluster Number** | **C1** | **C5** | **C2** | **C6** | **C3** | **C4** | **C7** | **C8** | **C9** | **C10** | **C11** | **C12** | **C13** |
| --- | --- | --- | --- | --- | --- | --- | --- | --- | --- | --- | --- | --- | --- |
| Patients, N | 1805 | 1096 | 1848 | 1209 | 1612 | 2323 | 1707 | 763 | 623 | 534 | 571 | 258 | 353 |
| Patients, % | 12.3 | 7.5 | 12.6 | 8.2 | 11.0 | 15.8 | 11.6 | 5.2 | 4.2 | 3.6 | 3.9 | 1.8 | 2.4 |
| **Chronic Conditions, %** |  |  |  |  |  |  |  |  |  |  |  |  |  |
| Lipid Metabolism Disorders | 87.5 | 87.9 | 78.1 | 85.6 | 77.2 | 79.3 | 89.8 | 66.2 | 68.2 | 68.4 | 71.3 | 100.0 | 0.0 |
| HTN | 95.9 | 95.2 | 84.3 | 85.1 | 78.2 | 82.9 | 87.7 | 67.8 | 71.8 | 61.4 | 100.0 | 0.0 | 0.0 |
| OA | 55.8 | 49.8 | 43.8 | 45.9 | 39.9 | 53.0 | 45.3 | 47.3 | 43.7 | 100.0 | 0.0 | 0.0 | 0.0 |
| Obesity | 58.2 | 42.2 | 29.1 | 51.9 | 28.7 | 48.0 | 38.0 | 38.9 | 100.0 | 0.0 | 0.0 | 0.0 | 0.0 |
| Behavioral Health | 41.5 | 30.9 | 56.1 | 36.9 | 31.6 | 37.3 | 27.5 | 100.0 | 0.0 | 0.0 | 0.0 | 0.0 | 0.0 |
| CVD | 77.8 | 57.7 | 68.5 | 3.2 | 41.5 | 51.0 | 100.0 | 0.1 | 0.0 | 0.0 | 0.0 | 0.0 | 0.0 |
| CPD | 55.7 | 34.2 | 38.3 | 14.1 | 24.1 | 99.0 | 0.1 | 0.1 | 0.0 | 0.0 | 0.0 | 0.0 | 0.0 |
| Cancer | 21.9 | 18.9 | 11.6 | 10.1 | 100.0 | 15.1 | 0.6 | 0.3 | 0.0 | 0.0 | 0.0 | 0.0 | 0.0 |
| Diabetes | 54.2 | 42.2 | 35.9 | 99.8 | 16.5 | 31.5 | 30.8 | 0.9 | 0.0 | 0.0 | 0.0 | 0.0 | 0.0 |
| Neurological Conditions | 18.8 | 1.7 | 94.5 | 2.1 | 16.3 | 1.3 | 0.2 | 1.3 | 0.0 | 0.0 | 0.0 | 0.0 | 0.0 |
| CKD | 36.3 | 99.9 | 24.8 | 1.0 | 2.5 | 3.4 | 0.3 | 3.9 | 0.0 | 0.0 | 0.0 | 0.0 | 0.0 |
| CHF | 98.6 | 9.4 | 19.4 | 0.4 | 5.8 | 1.7 | 0.2 | 0.0 | 0.0 | 0.0 | 0.0 | 0.0 | 0.0 |
