# Supplementary material for: A cluster-based approach for integrating clinical management of Medicare beneficiaries with multiple chronic conditions
Source: PLoS One. 2019 Jun 19;14(6):e0217696. doi: 10.1371/journal.pone.0217696 (PMC6584004; doi:10.1371/journal.pone.0217696)
Supplement: S6 Table — Cluster assignment in each randomly assigned group is shown in top 3 rows with mapping to the aggregated cluster in row 4. Cluster names in row 4 are assigned based on the most prevalent chronic condition in each of the 12 aggregated clusters. Abbreviations: HTN, hypertension; OA, osteoarthritis; CVD, cardiovascular disease; CPD, chronic pulmonary disease; CKD, chronic kidney disease; CHF, congestive heart failure; DM, diabetes mellitus; CA, cancer; BH, behavioral health; Obes, obesity. (DOCX) [file pone.0217696.s006.docx]

| Cluster, Group A | A1 | A2 | A4 | A6 | A3 | A7 | A5 | A8 | A9 | A10 | A11 | A12 | A13 |
| --- | --- | --- | --- | --- | --- | --- | --- | --- | --- | --- | --- | --- | --- |
| Cluster, Group B | B1 | B2 | B6 | B4 | B3 | B8 | B5 | B7 | B10 | B9 | B11 | B12 | B13 |
| Cluster, Group C | C1 | C5 | C2 | C6 | C3 | C4 | C7 | C8 | C9 | C10 | C11 | C12 | C13 |
| **Cluster Name** | **CHF** | **CKD** | **Neuro** | **DM** | **CA** | **CPD** | **CVD** | **BH** | **Obes** | **OA** | **HTN** | **HLP** | **Other** |
| Patients, N | 6044 | 4053 | 4176 | 5093 | 5099 | 4125 | 5730 | 3024 | 1879 | 1873 | 1736 | 794 | 1019 |
| Patients, % | 13.5 | 9.1 | 9.4 | 11.4 | 11.4 | 9.2 | 12.8 | 6.8 | 4.2 | 4.2 | 3.9 | 1.8 | 2.3 |
| **Chronic Conditions, %** |  |  |  |  |  |  |  |  |  |  |  |  |  |
| Lipid Metabolism Disorders | 89.7 | 87.3 | 73.8 | 84.6 | 79.5 | 75.8 | 88.7 | 68.2 | 69.5 | 69.5 | 70.0 | 100.0 | 0.0 |
| HTN | 96.3 | 95.6 | 80.9 | 87.6 | 80.0 | 78.3 | 87.6 | 68.6 | 72.8 | 65.2 | 100.0 | 0.0 | 0.0 |
| OA | 56.3 | 50.7 | 42.6 | 48.7 | 44.3 | 51.9 | 45.8 | 39.3 | 36.2 | 100.0 | 0.0 | 0.0 | 0.0 |
| Obesity | 55.6 | 39.8 | 30.4 | 49.3 | 32.3 | 41.1 | 44.1 | 39.4 | 100.0 | 15.1 | 0.0 | 0.0 | 0.0 |
| Behavioral Health | 50.2 | 33.3 | 50.7 | 35.5 | 30.1 | 26.3 | 30.9 | 99.8 | 0.0 | 0.0 | 0.0 | 0.0 | 0.0 |
| CVD | 80.7 | 59.1 | 58.7 | 23.0 | 42.7 | 38.6 | 99.8 | 8.4 | 6.4 | 0.0 | 0.0 | 0.0 | 0.0 |
| CPD | 59.9 | 33.2 | 34.5 | 22.3 | 28.9 | 99.4 | 22.4 | 22.2 | 0.0 | 0.0 | 0.0 | 0.0 | 0.0 |
| Cancer | 23.2 | 17.8 | 10.9 | 8.1 | 100.0 | 12.6 | 2.3 | 0.5 | 0.0 | 0.0 | 0.0 | 0.0 | 0.0 |
| Diabetes | 54.7 | 44.7 | 29.8 | 99.2 | 20.1 | 17.9 | 28.9 | 2.6 | 0.0 | 0.0 | 0.0 | 0.0 | 0.0 |
| Neurological Conditions | 26.9 | 15.8 | 97.5 | 6.2 | 10.9 | 0.9 | 4.0 | 0.9 | 0.0 | 0.0 | 0.0 | 0.0 | 0.0 |
| CKD | 36.5 | 99.9 | 12.6 | 1.6 | 7.0 | 2.4 | 0.5 | 1.0 | 0.0 | 0.0 | 0.0 | 0.0 | 0.0 |
| CHF | 94.8 | 13.2 | 10.9 | 0.8 | 6.1 | 1.1 | 0.9 | 1.0 | 0.0 | 0.0 | 0.0 | 0.0 | 0.0 |
