# Supplementary material for: A cluster-based approach for integrating clinical management of Medicare beneficiaries with multiple chronic conditions
Source: PLoS One. 2019 Jun 19;14(6):e0217696. doi: 10.1371/journal.pone.0217696 (PMC6584004; doi:10.1371/journal.pone.0217696)
Supplement: S7 Table — To evaluate the effect of ties in the distance data, the clustering analysis is repeated for several random permutations of patient order. Permutations are created by random re-ordering of patients, using those in subgroup A. The prevalence of conditions among patients in each cluster are shown here for the repeated analysis. Permutation #1 contains the results of patients in the order previously described in the paper, and permutations # 2 through # 6 are shown for five additional random orders. The cluster numbers are arranged for convenience in comparing the results. There is complete agreement in 3 clusters for all permutations, shown in the 3 last columns and highlighted in blue. Two additional clusters show identical results from permutations 2, 3 and 6 which are highlighted in yellow. Other examples where the prevalence of a particular condition is identical or differs slightly (<3%) in each permutation is highlighted in grey. For other chronic conditions, there is often close agreement in 5 of the 6 permutations. Overall, there is general agreement in the clustering solutions, especially when viewed in terms of burden of disease. However, there are differences that remain. Further analysis with various clustering methods and/or with additional populations may provide insight and confirmation of these results. Abbreviations: HTN, hypertension; OA, osteoarthritis; CPD, chronic pulmonary disease; CVD, cardiovascular disease; CKD, chronic kidney disease; CHF, congestive heart failure. (DOCX) [file pone.0217696.s007.docx]

| **Permutation #1 Clusters** | **1** | **2** | **4** | **3** | **5** | **7** | **6** | **8** | **9** | **10** | **11** | **12** | **13** |
| --- | --- | --- | --- | --- | --- | --- | --- | --- | --- | --- | --- | --- | --- |
| Lipid Metabolism Disorders | 90.5 | 88.1 | 72.2 | 78.8 | 89.0 | 74.4 | 82.1 | 66.1 | 72.4 | 69.1 | 70.0 | 100.0 | 0.0 |
| HTN | 95.8 | 95.6 | 79.2 | 80.6 | 87.3 | 72.7 | 87.2 | 66.8 | 75.3 | 64.8 | 100.0 | 0.0 | 0.0 |
| OA | 57.8 | 48.4 | 47.8 | 45.9 | 44.3 | 46.2 | 41.7 | 45.7 | 50.8 | 100.0 | 0.0 | 0.0 | 0.0 |
| Obesity | 53.1 | 35.8 | 34.2 | 38.3 | 47.3 | 28.6 | 44.9 | 43.1 | 100.0 | 0.0 | 0.0 | 0.0 | 0.0 |
| Behavioral Health | 50.5 | 36.4 | 44.9 | 29.0 | 44.1 | 2.9 | 32.5 | 99.5 | 0.0 | 0.0 | 0.0 | 0.0 | 0.0 |
| Diabetes | 55.5 | 43.9 | 29.7 | 22.9 | 34.5 | 0.5 | 100.0 | 0.6 | 0.0 | 0.0 | 0.0 | 0.0 | 0.0 |
| CPD | 60.1 | 35.2 | 33.8 | 25.0 | 28.2 | 99.8 | 28.7 | 32.7 | 0.0 | 0.0 | 0.0 | 0.0 | 0.0 |
| CVD | 81.6 | 63.2 | 49.8 | 38.9 | 100.0 | 36.2 | 20.9 | 0.4 | 15.0 | 0.0 | 0.0 | 0.0 | 0.0 |
| Cancer | 27.2 | 23.7 | 16.3 | 100.0 | 2.2 | 15.6 | 3.0 | 1.3 | 0.0 | 0.0 | 0.0 | 0.0 | 0.0 |
| Neurological Conditions | 26.2 | 24.3 | 99.9 | 4.8 | 8.3 | 0.3 | 3.7 | 0.9 | 0.0 | 0.0 | 0.0 | 0.0 | 0.0 |
| CKD | 33.5 | 99.8 | 1.9 | 2.2 | 1.0 | 1.9 | 1.8 | 0.0 | 0.0 | 0.0 | 0.0 | 0.0 | 0.0 |
| CHF | 94.7 | 17.3 | 5.5 | 2.1 | 1.5 | 0.5 | 0.5 | 2.8 | 0.0 | 0.0 | 0.0 | 0.0 | 0.0 |
| **Permutation #2 Clusters** | **12** | **13** | **4** | **6** | **9** | **8** | **11** | **10** | **7** | **5** | **3** | **2** | **1** |
| Lipid Metabolism Disorders | 89.9 | 88.9 | 64.9 | 80.5 | 87.6 | 77.8 | 87.7 | 65.8 | 68.9 | 70.5 | 70.0 | 100.0 | 0.0 |
| HTN | 95.6 | 95.4 | 76.8 | 82.9 | 84.5 | 77.1 | 89.2 | 71.1 | 68.4 | 68.7 | 100.0 | 0.0 | 0.0 |
| OA | 61.0 | 43.6 | 51.7 | 47.0 | 53.5 | 59.7 | 33.0 | 33.9 | 0.0 | 100.0 | 0.0 | 0.0 | 0.0 |
| Obesity | 49.0 | 47.4 | 25.3 | 37.8 | 43.6 | 43.3 | 47.3 | 36.1 | 100.0 | 36.2 | 0.0 | 0.0 | 0.0 |
| Behavioral Health | 49.9 | 32.1 | 46.9 | 25.0 | 44.4 | 23.0 | 27.0 | 99.3 | 0.0 | 0.0 | 0.0 | 0.0 | 0.0 |
| Diabetes | 45.5 | 53.2 | 9.7 | 34.1 | 15.0 | 18.7 | 96.7 | 12.2 | 0.0 | 0.0 | 0.0 | 0.0 | 0.0 |
| CPD | 61.5 | 33.2 | 41.1 | 20.1 | 14.3 | 99.5 | 27.2 | 26.8 | 0.0 | 0.0 | 0.0 | 0.0 | 0.0 |
| CVD | 80.7 | 62.4 | 52.5 | 44.3 | 100.0 | 35.5 | 43.3 | 7.5 | 0.0 | 0.0 | 0.0 | 0.0 | 0.0 |
| Cancer | 28.7 | 20.1 | 7.9 | 100.0 | 1.0 | 10.9 | 1.0 | 14.6 | 0.0 | 0.0 | 0.0 | 0.0 | 0.0 |
| Neurological Conditions | 27.1 | 10.5 | 98.8 | 16.6 | 8.0 | 0.5 | 18.7 | 3.8 | 0.0 | 0.0 | 0.0 | 0.0 | 0.0 |
| CKD | 34.3 | 99.9 | 21.0 | 5.2 | 0.1 | 1.6 | 0.9 | 1.6 | 0.0 | 0.0 | 0.0 | 0.0 | 0.0 |
| CHF | 95.6 | 16.6 | 4.9 | 2.9 | 1.3 | 0.3 | 3.3 | 1.5 | 0.0 | 0.0 | 0.0 | 0.0 | 0.0 |
| **Permutation #3 Clusters** | **10** | **13** | **7** | **1** | **6** | **5** | **4** | **11** | **9** | **8** | **3** | **12** | **2** |
| Lipid Metabolism Disorders | 91.0 | 82.2 | 80.2 | 80.4 | 86.4 | 69.0 | 87.0 | 64.6 | 68.9 | 70.5 | 70.0 | 100.0 | 0.0 |
| HTN | 95.6 | 93.3 | 86.1 | 82.0 | 85.1 | 70.3 | 89.8 | 63.2 | 68.4 | 68.7 | 100.0 | 0.0 | 0.0 |
| OA | 52.4 | 40.0 | 51.4 | 46.8 | 47.6 | 53.2 | 48.4 | 36.2 | 0.0 | 100.0 | 0.0 | 0.0 | 0.0 |
| Obesity | 50.1 | 28.2 | 41.9 | 34.0 | 31.0 | 38.6 | 56.6 | 43.1 | 100.0 | 36.2 | 0.0 | 0.0 | 0.0 |
| Behavioral Health | 43.7 | 13.6 | 68.9 | 32.2 | 6.8 | 21.0 | 31.2 | 99.4 | 0.0 | 0.0 | 0.0 | 0.0 | 0.0 |
| Diabetes | 50.5 | 28.9 | 31.9 | 29.3 | 3.6 | 0.0 | 99.3 | 0.2 | 0.0 | 0.0 | 0.0 | 0.0 | 0.0 |
| CPD | 59.2 | 25.2 | 40.0 | 34.5 | 32.7 | 99.9 | 31.3 | 22.8 | 0.0 | 0.0 | 0.0 | 0.0 | 0.0 |
| CVD | 81.4 | 48.7 | 70.4 | 47.6 | 100.0 | 0.0 | 43.6 | 2.0 | 0.0 | 0.0 | 0.0 | 0.0 | 0.0 |
| Cancer | 28.0 | 20.9 | 3.5 | 99.8 | 0.0 | 1.5 | 1.2 | 1.2 | 0.0 | 0.0 | 0.0 | 0.0 | 0.0 |
| Neurological Conditions | 20.9 | 7.6 | 71.0 | 13.8 | 0.2 | 0.3 | 1.1 | 0.2 | 0.0 | 0.0 | 0.0 | 0.0 | 0.0 |
| CKD | 31.3 | 100.0 | 29.2 | 10.4 | 0.7 | 1.8 | 4.7 | 0.0 | 0.0 | 0.0 | 0.0 | 0.0 | 0.0 |
| CHF | 96.2 | 18.1 | 13.8 | 3.6 | 0.2 | 0.0 | 3.2 | 0.4 | 0.0 | 0.0 | 0.0 | 0.0 | 0.0 |
| **Permutation #4 Clusters** | **3** | **8** | **7** | **9** | **10** | **5** | **11** | **4** | **6** | **13** | **2** | **12** | **1** |
| Lipid Metabolism Disorders | 90.5 | 85.4 | 67.8 | 80.1 | 86.6 | 68.9 | 86.6 | 77.6 | 74.6 | 69.1 | 70.0 | 100.0 | 0.0 |
| HTN | 96.1 | 95.4 | 76.9 | 81.2 | 81.2 | 68.4 | 89.4 | 76.8 | 77.3 | 64.8 | 100.0 | 0.0 | 0.0 |
| OA | 56.4 | 47.7 | 47.7 | 42.7 | 44.0 | 41.8 | 47.5 | 49.7 | 46.7 | 100.0 | 0.0 | 0.0 | 0.0 |
| Obesity | 49.2 | 37.1 | 31.1 | 31.2 | 0.0 | 45.7 | 53.6 | 48.9 | 100.0 | 0.0 | 0.0 | 0.0 | 0.0 |
| Behavioral Health | 43.3 | 33.6 | 56.1 | 34.2 | 0.0 | 24.9 | 37.0 | 100.0 | 0.0 | 0.0 | 0.0 | 0.0 | 0.0 |
| Diabetes | 56.6 | 41.9 | 7.5 | 26.8 | 0.0 | 9.0 | 99.7 | 0.0 | 0.0 | 0.0 | 0.0 | 0.0 | 0.0 |
| CPD | 64.3 | 23.9 | 36.2 | 32.0 | 31.6 | 99.7 | 29.6 | 28.6 | 0.0 | 0.0 | 0.0 | 0.0 | 0.0 |
| CVD | 82.5 | 58.3 | 50.4 | 47.5 | 100.0 | 15.0 | 46.9 | 40.2 | 26.7 | 0.0 | 0.0 | 0.0 | 0.0 |
| Cancer | 27.3 | 17.8 | 3.9 | 99.9 | 0.0 | 2.7 | 2.6 | 0.6 | 5.8 | 0.0 | 0.0 | 0.0 | 0.0 |
| Neurological Conditions | 28.8 | 20.9 | 99.9 | 14.2 | 3.2 | 1.7 | 13.2 | 1.1 | 0.0 | 0.0 | 0.0 | 0.0 | 0.0 |
| CKD | 40.7 | 99.9 | 2.4 | 5.1 | 0.8 | 2.6 | 2.1 | 2.2 | 0.0 | 0.0 | 0.0 | 0.0 | 0.0 |
| CHF | 92.0 | 12.8 | 4.0 | 4.0 | 0.0 | 3.7 | 1.1 | 7.0 | 0.2 | 0.0 | 0.0 | 0.0 | 0.0 |
| **Permutation #5 Clusters** | **9** | **13** | **3** | **2** | **7** | **5** | **8** | **10** | **11** | **4** | **6** | **1** | **12** |
| Lipid Metabolism Disorders | 88.8 | 86.4 | 76.5 | 79.4 | 87.0 | 65.6 | 83.0 | 78.4 | 65.6 | 77.7 | 70.0 | 100.0 | 0.0 |
| HTN | 96.0 | 95.3 | 82.1 | 80.2 | 84.7 | 67.3 | 90.5 | 73.6 | 68.3 | 75.3 | 100.0 | 0.0 | 0.0 |
| OA | 53.1 | 53.2 | 46.0 | 46.7 | 49.4 | 45.8 | 29.2 | 46.5 | 0.7 | 100.0 | 0.0 | 0.0 | 0.0 |
| Obesity | 44.6 | 42.0 | 42.6 | 31.5 | 28.2 | 42.0 | 56.6 | 53.6 | 100.0 | 43.2 | 0.0 | 0.0 | 0.0 |
| Behavioral Health | 43.4 | 33.1 | 57.2 | 32.0 | 29.2 | 36.5 | 22.0 | 99.3 | 0.0 | 0.0 | 0.0 | 0.0 | 0.0 |
| Diabetes | 51.9 | 40.3 | 35.9 | 23.5 | 8.6 | 0.6 | 99.7 | 35.7 | 0.0 | 29.7 | 0.0 | 0.0 | 0.0 |
| CPD | 65.3 | 34.2 | 37.4 | 26.3 | 31.2 | 99.9 | 50.5 | 0.9 | 1.4 | 0.0 | 0.0 | 0.0 | 0.0 |
| CVD | 80.9 | 60.3 | 58.0 | 42.1 | 100.0 | 0.8 | 45.4 | 21.1 | 0.0 | 6.5 | 0.0 | 0.0 | 0.0 |
| Cancer | 30.7 | 20.0 | 13.2 | 99.9 | 1.4 | 0.1 | 2.1 | 0.1 | 0.0 | 0.0 | 0.0 | 0.0 | 0.0 |
| Neurological Conditions | 21.3 | 18.7 | 99.9 | 7.8 | 6.0 | 0.5 | 1.8 | 0.1 | 4.8 | 0.0 | 0.0 | 0.0 | 0.0 |
| CKD | 32.1 | 99.3 | 13.3 | 6.0 | 0.1 | 0.6 | 1.2 | 2.1 | 0.0 | 0.0 | 0.0 | 0.0 | 0.0 |
| CHF | 90.4 | 11.8 | 16.3 | 3.0 | 1.0 | 2.3 | 0.8 | 0.1 | 0.0 | 0.0 | 0.0 | 0.0 | 0.0 |
| **Permutation #6 Clusters** | **11** | **4** | **12** | **1** | **3** | **13** | **6** | **8** | **10** | **5** | **2** | **9** | **7** |
| Lipid Metabolism Disorders | 89.7 | 84.5 | 74.5 | 80.5 | 87.0 | 71.1 | 86.1 | 82.3 | 68.9 | 70.5 | 70.0 | 100.0 | 0.0 |
| HTN | 96.6 | 93.3 | 81.0 | 81.2 | 83.9 | 71.1 | 88.3 | 82.8 | 68.4 | 68.7 | 100.0 | 0.0 | 0.0 |
| OA | 57.0 | 47.2 | 39.9 | 45.1 | 44.3 | 48.4 | 37.0 | 57.9 | 0.0 | 100.0 | 0.0 | 0.0 | 0.0 |
| Obesity | 50.0 | 38.1 | 25.9 | 37.4 | 41.4 | 29.0 | 48.5 | 61.6 | 100.0 | 36.2 | 0.0 | 0.0 | 0.0 |
| Behavioral Health | 49.6 | 28.7 | 63.4 | 23.7 | 0.0 | 19.9 | 0.0 | 93.6 | 0.0 | 0.0 | 0.0 | 0.0 | 0.0 |
| Diabetes | 59.1 | 29.1 | 23.9 | 35.9 | 0.2 | 7.1 | 100.0 | 40.5 | 0.0 | 0.0 | 0.0 | 0.0 | 0.0 |
| CPD | 51.6 | 31.0 | 38.0 | 29.6 | 11.9 | 100.0 | 18.6 | 37.8 | 0.0 | 0.0 | 0.0 | 0.0 | 0.0 |
| CVD | 79.9 | 55.9 | 66.5 | 38.9 | 100.0 | 34.5 | 37.9 | 34.1 | 0.0 | 0.0 | 0.0 | 0.0 | 0.0 |
| Cancer | 19.4 | 23.1 | 21.2 | 100.0 | 0.0 | 11.8 | 0.1 | 0.8 | 0.0 | 0.0 | 0.0 | 0.0 | 0.0 |
| Neurological Conditions | 31.2 | 6.8 | 80.7 | 5.2 | 1.0 | 2.1 | 0.6 | 2.6 | 0.0 | 0.0 | 0.0 | 0.0 | 0.0 |
| CKD | 47.6 | 99.8 | 5.4 | 4.3 | 0.0 | 0.1 | 0.1 | 4.8 | 0.0 | 0.0 | 0.0 | 0.0 | 0.0 |
| CHF | 85.4 | 11.7 | 8.1 | 7.7 | 0.2 | 3.2 | 0.1 | 0.4 | 0.0 | 0.0 | 0.0 | 0.0 | 0.0 |
